# Comparison of Subgingival and Buccal Mucosa Microbiome in Chronic and Aggressive Periodontitis: A Pilot Study

**DOI:** 10.3389/fcimb.2019.00053

**Published:** 2019-03-11

**Authors:** Yiping Wei, Meng Shi, Min Zhen, Cui Wang, Wenjie Hu, Yong Nie, Xiaolei Wu

**Affiliations:** ^1^National Engineering Laboratory for Digital and Material Technology of Stomatology, Beijing Key Laboratory of Digital Stomatology, Department of Periodontology, National Clinical Research Center for Oral Diseases, Peking University School and Hospital of Stomatology, Beijing, China; ^2^Laboratory of Environmental Microbiology, Department of Energy and Resources Engineering, College of Engineering, Peking University, Beijing, China

**Keywords:** 16S sequencing, subgingival microbiome, buccal mucosa microbiome, chronic periodontitis, aggressive periodontitis

## Abstract

Periodontal microorganisms not only colonize subgingival pockets, but also are detected on various mucous membranes in patients with periodontitis. The object of this pilot study was, using the next-generation sequencing of 16S RNA gene, to characterize the microbiota in two oral habitats (buccal mucosas and subgingival pockets) in patients with different forms of periodontitis. Thirty-two buccal swab samples and 113 subgingival samples were obtained from eleven subjects with chronic periodontitis (ChP), twelve subjects with aggressive periodontitis (AgP), and nine periodontally healthy individuals (HP). Using Miseq Sequencing of 16S rRNA gene, we found that the subgingival and buccal mucosa microbiome of ChP and AgP patients both differed from HP. Meanwhile, *Veillonella, Treponema, Filifactor, Fretibacterium, Peptostreptococcaceae_[XI][G-6], Peptostreptococcaceae_[XI][G-5], Bacteroidetes_[G-5], Bacteroidetes_[G-3], Peptostreptococcaceae_[XI][G-4], Peptostreptococcaceae_[XI][G-2]* significantly increased both in buccal and subgingival plaque samples in periodontitis subjects (ChP and AgP) compared with HP. Moreover, the results based on the Unweighted UniFrac distance showed that buccal and subgingival plaque samples from the same individuals show higher community divergence than same habitats from different subject samples. This study demonstrated that the microbiome of buccal mucosa can be influenced by periodontitis. However, subgingival and buccal mucosa microbiome seem to be characterized by species-specific colonization patterns. This pilot study provides a glimpse at the changes of subgingival and buccal mucosa associated with periodontitis from a holistic view. Further studies should be taken to illuminate the interplay between these detected changes and periodontitis development.

## Introduction

The bacteria colonizing the hard and soft tissues of the oral cavity profoundly influence oral health and disease. Microbiologic studies have identified over 700 species of microorganisms in oral cavity (Aas et al., 2005), including a large number of uncultivable species (~50%), more than 400 of which have been detected in the subgingival crevice/pocket (Paster et al., 2006), the remaining species have been identified from other oral habitats such as the tongue, oral mucous membranes, and carious lesions.

Periodontitis is a chronic inflammatory disease initiated by colonization of subgingival periodontal pathogens. It has been shown that periodontal microorganisms are not only restricted to subgingival pockets, but are also found on various mucous membranes in patients with periodontitis (Van Winkelhoff et al., 1986; Eger et al., 1996; Gohler et al., 2018). Several studies have shown that buccal mucosa surfaces harbor periodontal pathogens (Danser et al., 1996; Socransky et al., 1999), and have suggested that it may serve as reservoirs for infection or reinfection of the periodontal tissues and deserve therapeutic attention (Zambon et al., 1981; Quirynen et al., 2001).

Aggressive periodontitis (AgP) is recognized as a group of periodontal diseases with faster destruction of connective tissue attachment that are different from chronic periodontitis (ChP) (Lang et al., 1999). Decades of studies have tried to distinguish AgP from ChP, however, the notion that AgP has a different microbiological pathogenesis from ChP has still not been confirmed. Notably, several studies have found that sampling oral soft tissues was a reliable method for monitoring the presence of A. actinomycetemcomitans (Müller et al., 1995; Eger et al., 1996), which has been considered to be closely related to the development of periodontitis, especially the development of AgP.

Microbiota on buccal mucosa can be collected easily, non-invasively, and repetitively. Therefore, sampling the microbiota on oral mucosa seems to be a promising way to diagnose periodontal diseases. Some investigations have demonstrated that the buccal mucosa microbiota in periodontally healthy and periodontitis subjects differ, but almost were based on traditional identification of bacteria in cultivations or culture-independent molecular studies. Recently, the next-generation sequencing (NGS) of bacterial 16S rRNA gene makes it possible to broaden our knowledge of the complexity of the microbiome, which has the advantage of detecting the composition of microbial communities in unprecedented depth. Several studies using NGS were to assess the composition of the polymicrobial periodontal community (Liu et al., 2012; Abusleme et al., 2013; Szafranski et al., 2015; Schulz et al., 2019). However, there is scarce evidence on microbiota that colonize the buccal mucosa of different forms of periodontitis.

Thus, the primary objective of this study was, using NGS of the16S RNA gene, to characterize the microbiota in two oral habitats (buccal mucosas and subgingival pockets) in patients with ChP or AgP, and to compare them with samples of subjects with periodontal health (HP).

## Materials and Methods

### Study Population and Clinical Examination

From January 2017 to July 2017, eleven patients with ChP and twelve patients with AgP were recruited from the Department of Periodontology, Peking University School and Hospital of Stomatology, and nine HP subjects were selected as controls. The clinical inclusion criteria for all participants were that: (1) they agreed to join the research and signed an informed consent, (2) they were free of systemic disease and not pregnant or lactating, (3) they had not received any kind of periodontal treatment in the past half year, nor had taken antibiotics within the past 3 months, (4) without removable or fixed denture in month, (5) without salivary glands disease, (6) without aggressive caries, untreated pulp disease or periapical problems, (7) they were non-smoker. The clinical inclusion criteria for periodontally healthy individuals and the diagnostic criteria for ChP and AgP have been previously described in detail (Shi et al., 2018).

Full-mouth clinical examinations (including PD, AL, and BOP at six sites per tooth) were carried out by one practitioner (WH). His reliability was calibrated as described by Shi (Shi et al., 2018). Full-mouth periapical radiographs were taken as diagnostic basis.

The study protocol was conducted in accordance with the Declaration of Helsinki, and approved by the Ethics Committee of the Peking University Health Science Center (approval number: PKUSSIRB-201631135). All enrolled individuals gave written informed consent.

### Sample Collection

Buccal and subgingival plaque samples were collected 1 week after the full-mouth periodontal examination. All participants were requested to refrain from food for 8 h and oral hygiene (brushing or flossing the teeth) for 12 h before sampling. Samples from all subjects were collected and stored at −80°C for subsequent processing.

The buccal swab sample was collected before the subgingival plaque sample. Buccal swab sample was obtained from mucosa of both cheeks with a sterile cotton swab and placed in a separate sterile 1.5 ml Eppendorf microcentrifuge tube containing 1 ml TE buffer (50 mM Tris-HCl, 1 mM EDTA; pH 8.0). All subgingival plaque samples were collected from each participant from mesiobuccal sites of molars. For each patient with periodontitis, three to five samples were collected from the mesiobuccal site of molars with PD ≥ 4 mm. For each HP subject, two subgingival plaque samples were collected from the mesiobuccal site of molars with PD ≤ 3 mm without AL and BOP. The samples were obtained and processed was based on our previous research (Shi et al., 2018).

### Extraction of Total Genomic DNA

Total DNA was extracted from the buccal and subgingival plaque using a QIAamp DNA Mini Kit (QIAGEN Sciences, USA) following the manufacturer's instructions, with an extra lysozyme treatment (3 mg/mL, 1.5 h) for bacterial cell lysis. The final concentration was measured with a NanoDrop ND-1000 spectrophotometer (Thermo Fisher Scientific, USA) and monitored on 1% agarose gels. The results showed that the A260:A280 ratios were 1.8–2.1 and the DNA concentrations were all more than 20 ng/μL. Based on the quantity and the quality of the DNA extracted, samples were diluted to 1 ng/μL using sterile water and stored at −80°C until use.

### PCR Amplification and Sequencing Analysis

PCR amplification of the 16S ribosomal RNA gene V4-V5 region was performed using primers 338F (5', ACTCCTACGGGAGGCAGCAG, 3') and 806R (5', GGACTACNNGGGTATCTAAT, 3'), where barcode is an eight-base sequence unique to each sample. The PCR cycling conditions were used: initial temperature of 95°C for 3 min, followed by 25 cycles consisting of denaturation at 95°C for 30 s, annealing at 55°C for 30 s, and elongation at 72°C for 45 s and a final extension step at 72°C for 10 min. PCR reactions were performed in triplicate 20 μL mixture containing 4 μL of 5 × FastPfu Buffer, 2 μL of 2.5 mM dNTPs, 0.8 μL of each primer (5 μM), 0.4 μL of FastPfu Polymerase, and 10 ng of template DNA.PCR products were separated by 2% agarose gels electrophoresis, and purified using the AxyPrep DNA Gel Extraction Kit (Axygen Biosciences, Union City, CA, U.S.) according to the manufacturer's instructions and quantified with a QuantiFluor™ -ST Handheld Fluorometer with UV/Blue Channel (Promega, U.S.).

Purified amplicons were pooled in equimolar and paired-end sequenced (2 × 250) on an Illumina MiSeq platform at Majorbio Bio-Pharm Technology (Shanghai, China). Raw sequencing data was filtered and trimmed by using QIIME (version 1.17), and classified into operational taxonomic units (OTUs) with 97% similarity cutoff using UPARSE (version 7.1 http://drive5.com/uparse/). The taxonomy of each 16S rRNA gene sequence was analyzed by Ribosomal Database Project Classifier tool (http://rdp.cme.msu.edu/) against the Human Oral Microbiome Database using a default confidence threshold of 0.7 (Dewhirst et al., 2010). The raw reads were deposited into the NCBI Sequence Read Archive (SRA) database (Accession Number: SRP173111).

### Bioinformatic Analysis, Statistical Analysis, and Visualization

Normality tests were conducted in each group of data. The mean clinical parameters were then compared via a paired Student's *t*-test (PD, AL) or Mann–Whitney *U*-test (BI). After rarefying the OTU table, two metrics were calculated for the evaluation of alpha diversity: abundance-based-coverage (ACE) estimates the species abundance; and the diversity of the sample microbiota was estimated by the Shannon index. The Mann–Whitney *U* test was used to compare significant differences of the alpha diversity indexes between the different groups (*p* < 0.05). For evaluating the similarity of microbial community structure among all samples, a principal coordinates analysis (PCoA) was performed on the OTU level. Analysis of similarities was calculated to compare the intra- and inter-group similarities based on the Unweighted UniFrac distance, a phylogenetic measures of beta diversity which was calculated by QIIME. The Mann–Whitney *U*-test was used to analyze the differences between the Unweighted UniFrac distances among groups and the results were visualized by constructing a scatter diagram and box plot. PCoA analysis, scatter diagram and box plot and ternary plots were performed using R 3.2.5. The taxonomy compositions and abundances of different samples were analyzed and visualized by GraphPad PRISM® software (version 4.0). To compare the taxa in buccal and subgingival plaque samples between HP, AgP, and ChP, the Mann–Whitney *U*-test was used. The Mann–Whitney test, Student's *t*-test were performed using SPSS 20.0.

## Results

### Increasing Diversity in Buccal and Subgingival Microbiota of Patients With Periodontitis

Thirty-two subjects were recruited in the study, including twenty-three patients with periodontitis: eleven of them were diagnosed as ChP, twelve of them were diagnosed as AgP, and other nine subjects were defined as HP. Consequently, 32 buccal swab samples (showed as HP/ChP/AgP_B), and 113 subgingival samples (showed as HP_Sub: 1-2 sample(s) per/subject, ChP/AgP_Sub: 3-5 samples per/patient, for a total of 16 samples belonged to HP_Sub, 46 samples belonged to ChP_Sub and 51 samples belonged to AgP_Sub) were collected. The demographic and clinical metrics of subjects on baseline are detailed in Table 1. As expected, BOP, PD, and AL values were greater in the group with periodontitis than for HP subjects. Sequences were clustered to 959 OTUs at a 97% similarity level. A total of 12 phyla, 31 classes, 56 orders, 103 families, and 195 genera were detected (Table 2).

**Table 1:** Clinical Characteristic in patients with periodontitis and healthy subjects.

	**AgP (*n* = 12)**	**ChP (*n* = 11)**	**HP (*n* = 9)**
Age (years)	29.42 ± 4.23	38.18 ± 5.87	23.33 ± 1.65
**GENDER**			
Male	3	7	3
Female	9	4	6
**CLINICAL INDEX**			
PD (mm, full month)	4.7 ± 1.1	4.3 ± 0.7	2.4 ± 0.2
BI	3.8 ± 0.3	3.7 ± 0.8	0.4 ± 0.3
AL (mm, full month)	4.0 ± 0.7	3.7 ± 0.2	0

*Values are means ± standard deviations. PD, probing depth; BI, bleeding index; AL, attachment loss*.

**Table 2:** Number of raw tags, final tags, and OTUs of each sample by 16S rRNA sequencing.

**Sample id**	**Raw tags**	**Final tags**	**OTU**	**Sample id**	**Raw tags**	**Final tags**	**OTU**
A1_B	36,075	31,867	257	A3_b3	47,373	33,983	306
A2_B	39,322	36,330	280	A3_c2	44,545	33,204	361
A3_B	39,474	37,598	246	A4_a1	54,481	32,445	359
A4_B	31,854	30,811	245	A4_b1	68,686	36,632	445
A5_B	43,099	42,474	334	A4_c1	43,819	35,300	293
A6_B	33,307	32,927	218	A5_c1	82,047	45,239	416
A7_B	30,296	29,791	244	A5_c2	50,230	34,623	394
A8_B	37,190	33,840	335	A6_b2	37,141	23,961	359
A9_B	34,568	33,943	304	A6_b3	40,434	27,527	346
A10_B	35,986	31,822	175	A6_b4	56,828	38,003	405
A11_B	42,592	39,932	316	A6_c2	52,270	36,552	397
A12_B	36,922	34,488	320	A7_a1	37,400	28,941	314
C1_B	36,844	35,142	277	A7_a3	48,340	32,398	375
C2_B	34,084	33,048	232	A7_b2	48,436	35,754	371
C3_B	39,386	35,321	196	A7_b3	52,271	35,905	389
C4_B	40,932	38,466	301	A7_c2	51,190	36,538	365
C5_B	36,374	35,945	304	A8_a1	35,824	26,574	347
C6_B	39,888	39,283	246	A8_a2	56,042	54,965	418
C7_B	39,623	38,714	259	A8_b1	33,130	21,155	294
C8_B	43,558	42,018	265	A8_b2	30,404	21,736	345
C9_B	39,812	39,033	386	A9_a1	56,639	35,146	383
C10_B	36,365	34,882	221	A9_b1	60,387	39,335	328
C11_B	41,582	35,269	167	A9_b2	51,255	34,434	360
H1_B	34,097	29,249	226	A9_c1	58,851	38,920	326
H2_B	37,026	35,989	207	A9_c2	60,975	40,728	408
H3_B	30,144	29,067	117	A10_a1	42,800	31,507	306
H4_B	43,840	39,330	160	A10_a2	39,057	28,737	232
H5_B	33,012	31,325	206	A10_b1	38,224	27,662	278
H6_B	42,829	39,564	212	A10_b2	43,685	28,446	346
H7_B	35,998	34,676	233	A10_c1	57,706	36,449	331
H8_B	38,596	37,015	161	A10_c2	31,041	27,855	282
H9_B	35,736	34,726	110	A11_a1	54,024	51,443	450
A1_b1	46,552	35,254	177	A11_a2	48,279	35,087	345
A1_b2	33,488	26,616	216	A11_b1	43,927	29,257	327
A1_c1	60,964	39,076	353	A11_b2	49,050	35,600	303
A2_a1	43,844	30,686	221	A11_c1	49,958	35,461	318
A2_a2	35,787	27,181	203	A12_a1	52,131	33,801	373
A2_a3	49,611	33,318	199	A12_b1	54,516	36,065	379
A2_c1	32,990	27,002	292	A12_b2	48,430	33,457	372
A2_c2	39,841	30,296	226	A12_c1	54,024	34,815	405
A3_a2	39,708	27,677	331	A12_c2	52,473	34,206	397
A3_b1	51,841	35,266	357	C1_a1	41,030	28,836	217
C1_b1	35,973	33,693	206	C9_a1	43,329	34,184	313
C1_b2	66,129	59,736	197	C9_b1	41,179	32,085	343
C2_a2	40,684	30,412	272	C9_b2	40,897	28,088	343
C2_b2	43,279	35,034	218	C9_c1	39,799	34,116	324
C2_b3	40,113	32,670	256	C9_c2	54,060	34,074	370
C2_b4	30,175	24,869	206	C10_a1	47,987	31,874	348
C2_c2	44,509	32396	243	C10_a2	38668	36,074	261
C3_a2	41,227	37,297	339	C10_b1	42,563	28,810	303
C3_b1	57,180	33,541	415	C10_b2	46,520	34,064	269
C3_b3	37,500	34222	349	C11_a1	35,897	29,739	226
C3_c2	44,471	28,496	370	C11_a2	47,157	34,367	235
C4_b1	39,296	27,507	273	C11_b1	52,553	36,186	282
C4_c1	30,985	25,687	251	C11_b2	42,344	35,642	211
C4_c2	59,029	33,744	340	C11_c1	36,367	31,273	235
C5_a2	33,443	26,117	369	H2_d1	41,302	30,605	296
C5_b2	34,582	25,268	366	H2_d2	38,940	27,060	257
C5_b3	44,746	30,572	387	H3_d1	40,700	28,094	242
C5_c2	45,143	33,928	365	H3_d2	47,243	35,944	209
C6_a1	76,870	41,110	394	H4_d1	48,244	32,282	312
C6_b1	48,539	32,687	358	H4_d2	77,569	61,112	312
C6_b2	38,773	32,200	360	H5_d1	49,987	33,109	306
C6_c1	41,034	30,158	351	H5_d2	42,070	30,764	270
C7_a1	47,922	40,246	309	H6_d1	41,383	31,139	305
C7_a2	50,634	30,481	333	H6_d2	36,876	28,426	351
C7_b1	48,860	35,601	303	H7_d1	42,713	33,094	270
C7_b2	45,790	25,192	317	H7_d2	42,858	32,091	294
C8_a1	34,539	24,521	290	H8_d1	45,131	30,014	219
C8_a2	40,749	31,775	347	H8_d2	62,481	36,312	335
C8_a3	52,428	33,141	315	H9_d1	44,693	29,478	327
C8_b1	44,530	33,922	365	H9_d2	42,612	30,666	324
C8_b2	48,761	32,203	262				

**Figure 1:**
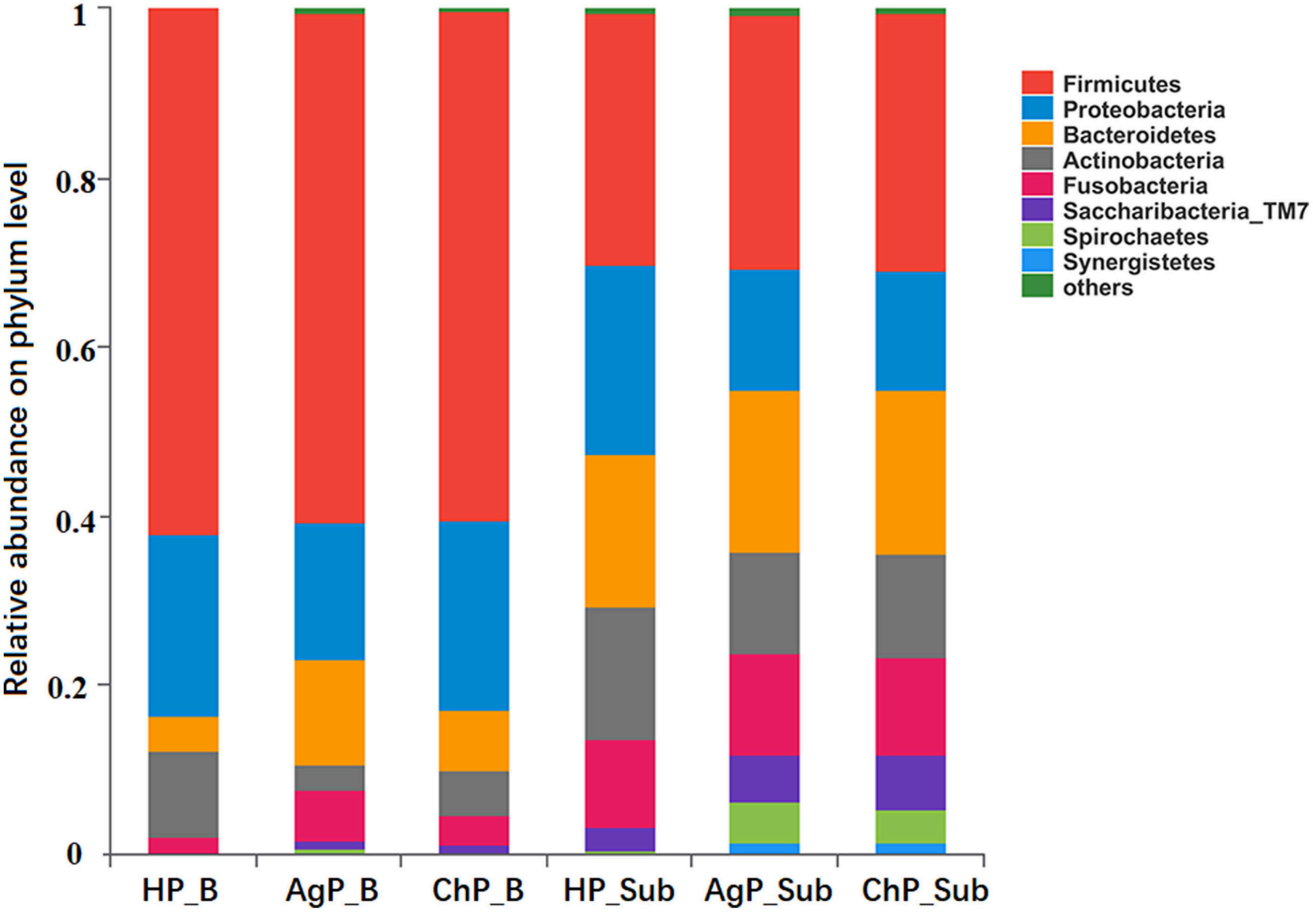
Relative abundance of bacterial composition on phylum level of buccal (shown as B) and subgingival plaque (shown as Sub) samples in health group (HP), chronic periodontitis group (ChP), and aggressive periodontitis group (AgP).

In the HP_B, ChP_B, and AgP_B groups, the five most abundant of the 12 phyla were Firmicutes, Proteobacteria, Bacteroidetes, Actinobacteria, and Fusobacteria, which represented more than 95% of the total sequences. In samples of subgingival plaque, HP_Sub, ChP_Sub, and AgP_Sub, the aforementioned five phyla also constituted the majority of the sequences, but the abundance of TM7, Spirochaetes, and Synergistetes increased, especially in ChP_Sub and AgP_Sub (Figure 1).

**Figure 2:**
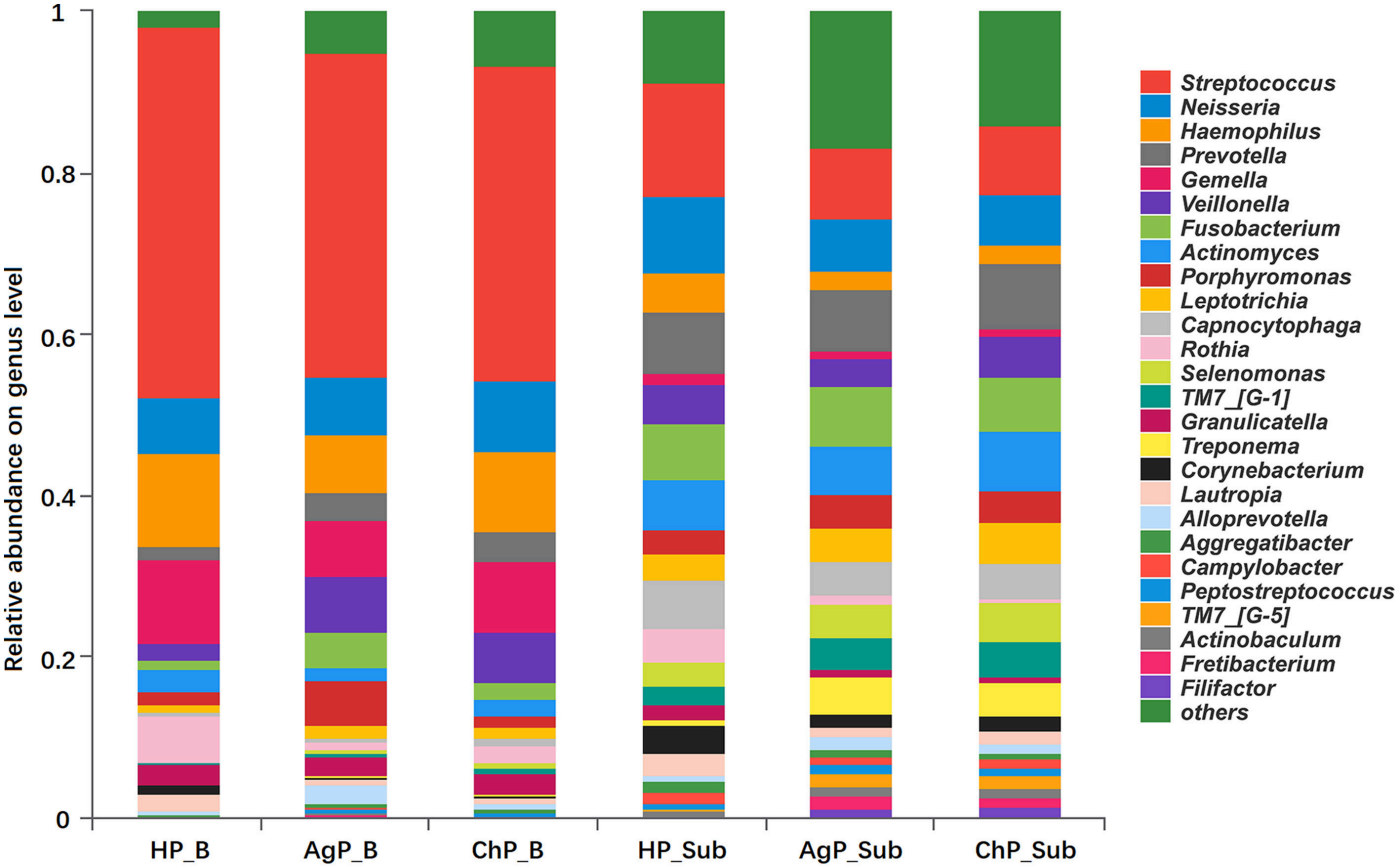
Relative abundance of bacterial composition on genus level of buccal (shown as B) and subgingival plaque (shown as Sub) samples in health group (HP), chronic periodontitis group (ChP), and aggressive periodontitis group (AgP).

At the genus level, among the 195 genera detected, the five most abundant in buccal samples were *Streptococcus, Haemophilus, Gemella, Neisseria, and Veillonella*. These constituted more than 60% of the total sequences. In subgingival plaque samples, *Streptococcus, Prevotella, Neisseria, Fusobacterium, Actinomyces, Capnocytophaga, Veillonella, and Leptotrichia* represented more than 50% of the total sequences (Figure 2).

**Figure 3:**
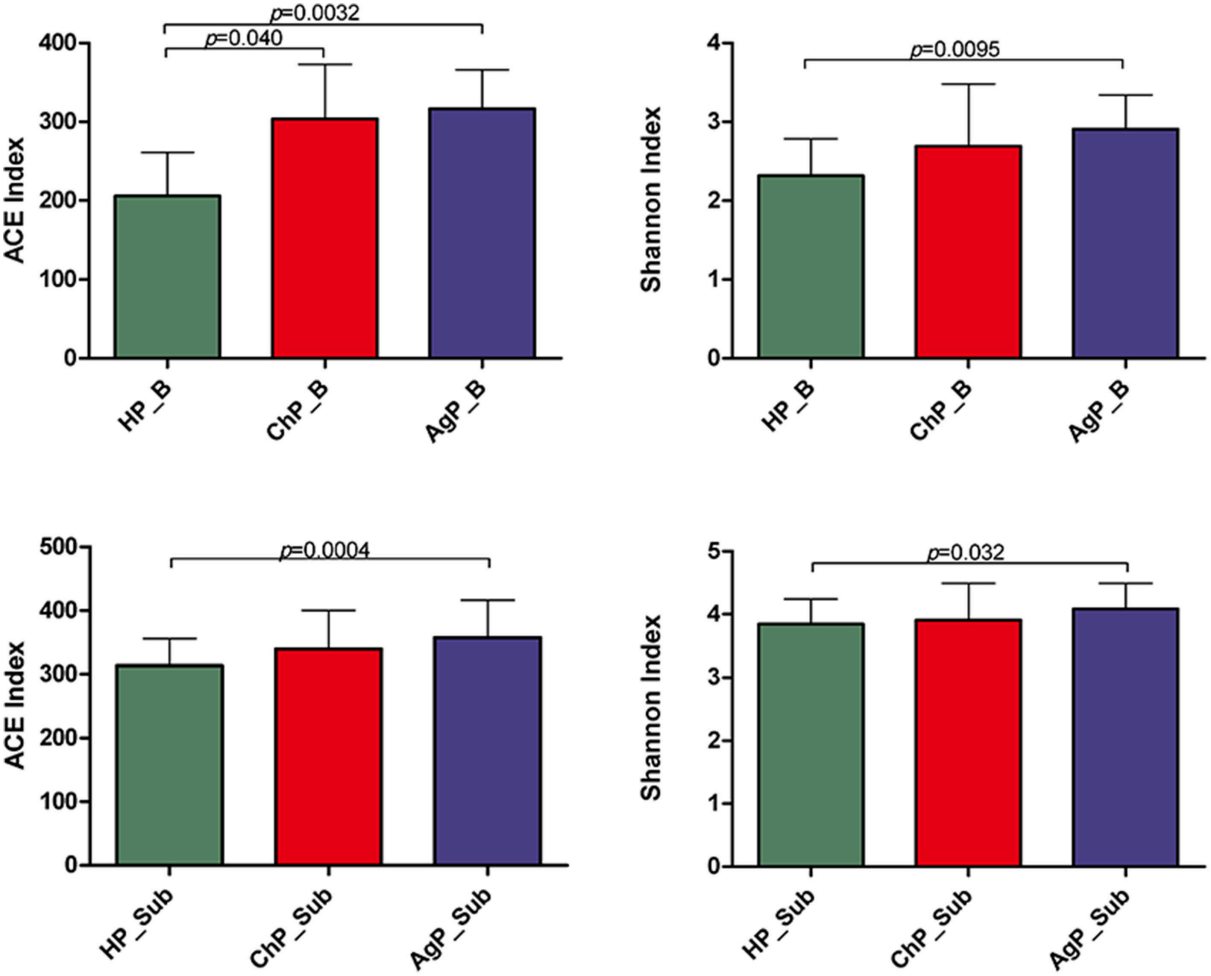
Bacterial community alpha-diversity as calculated by ACE and Shannon index of buccal (shown as B) and subgingival plaque (shown as Sub) samples in health group (HP), chronic periodontitis group (ChP), and aggressive periodontitis group (AgP). The error bars indicate mean with standard error.

The richness of the total amount of bacteria was estimated by ACE index. The diversity of the microbiota was estimated by Shannon index (Table 3). The ACE index in buccal microbiota of periodontitis patients compared to that of HP_B samples was significantly increased. The Shannon index was also higher in ChP_B and AgP_B, although there were no statistically significant differences among HP_B and ChP_B (Figure 3). As for HP_Sub, ChP_Sub, and AgP_Sub, ACE index and Shannon index had identical trend with the buccal samples. The results showed that the alpha-diversity of subjects with aggressive periodontitis was greater than that in healthy individuals, both in buccal and subgingival plaque samples.

**Table 3:** Summary of MiSeq sequencing data in each group.

**Group**	**OTUs**	**ACE index**	**Shannon index**
HP_B	181.33 ± 46.05	205.99 ± 55.33	2.32 ± 0.47
ChP_B	259.45 ± 59.20	303.90 ± 68.88	2.69 ± 0.79
AgP_B	272.83 ± 50.34	316.64 ± 49.68	2.90 ± 0.44
HP_Sub	289.31 ± 41.28	313.63 ± 42.32	3.85 ± 0.40
ChP_Sub	304.48 ± 59.44	339.83 ± 60.56	3.91 ± 0.59
AgP_Sub	335.75 ± 64.53	357.82 ± 58.66	4.09 ± 0.41

*The number of reads, OTUs, richness estimator ACE, and diversity estimator Shannon were calculated at the 97% similarity level. Values are means ± standard deviations*.

**Figure 4:**
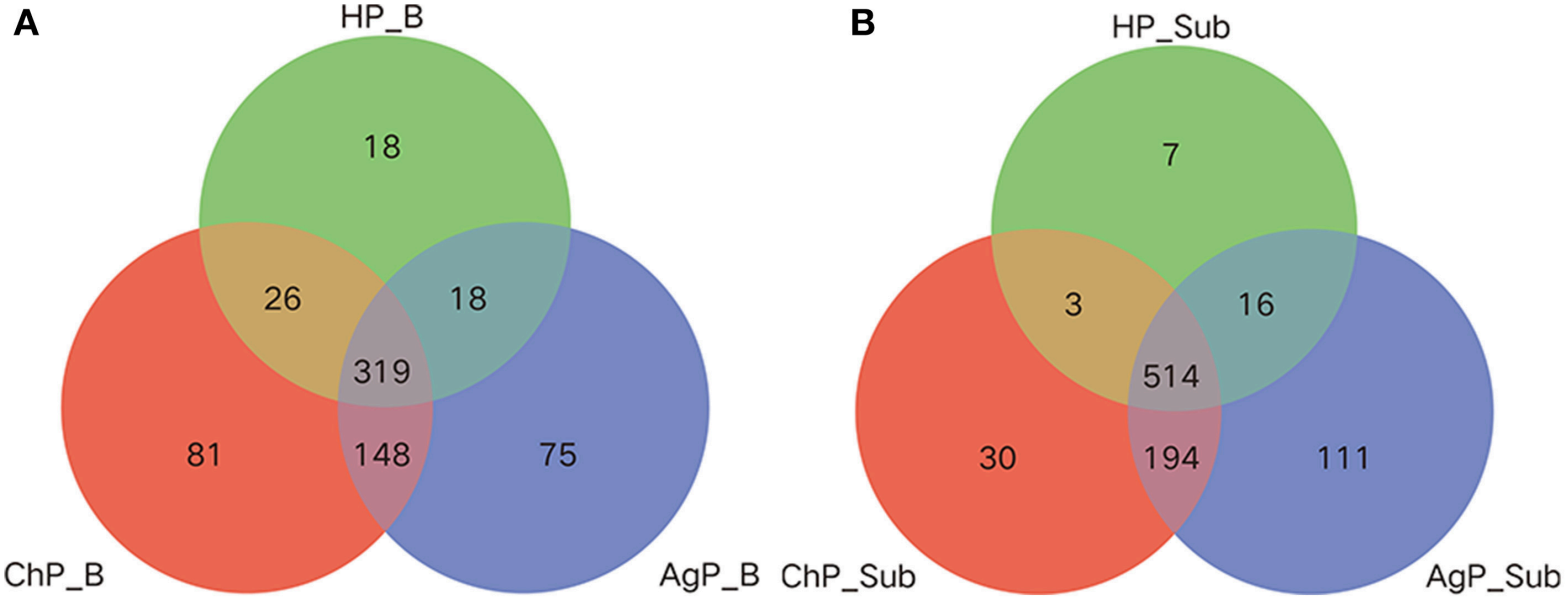
Venn diagram of the number of different OTUs common/unique to the buccal (shown as B) and subgingival plaque (shown as Sub) samples in health group (HP), chronic periodontitis group (ChP), and aggressive periodontitis group (AgP). The overlapping areas represent the number of OTUs shared by the counterpart samples. **(A)** A total of 685 kind of OTUs were detected in buccal samples of three groups. Three hundred nineteen kind of OTUs were shared by the HP_B, AgP_B, and ChP_B groups, while 18, 75, and 81 kind of OTUs were unique to the respective groups. **(B)** A total of 875 kind of OTUs were detected in the subgingival plaque samples of three groups. Five hundred fourteen kind of OTUs were shared by the HP_Sub, AgP_Sub, and ChP_Sub groups, and 7, 111, and 30 kind of OTUs were unique to the respective groups.

Venn diagram was used to show the number of different OTUs common/unique to the buccal and subgingival plaque samples in three groups. In the HP_B, ChP_B, and AgP_B groups, 319, 345, 337, 467 OTUs shared by samples HP_B/ChP_B/AgP_B, HP_B/ChP_B, HP_B/AgP_B, ChP_B/AgP_B, respectively. Measurements of unique OTUs in the HP_B, ChP_B, and AgP_B groups were 18, 81, and 75, respectively. As in the HP_Sub, ChP_Sub, and AgP_Sub groups, 514, 517, 530, 708 OTUs shared by samples HP_Sub/ChP_Sub/AgP_Sub, HP_Sub/ChP_Sub, HP_Sub/AgP_Sub, ChP_Sub/AgP_Sub, respectively. Measurements of unique OTUs in the HP_Sub, ChP_Sub and AgP_Sub groups were 7, 30, and 111, respectively (Figure 4).

### Comparison of Buccal and Subgingival Microbiota Between Health and Periodontitis

**Figure 5:**
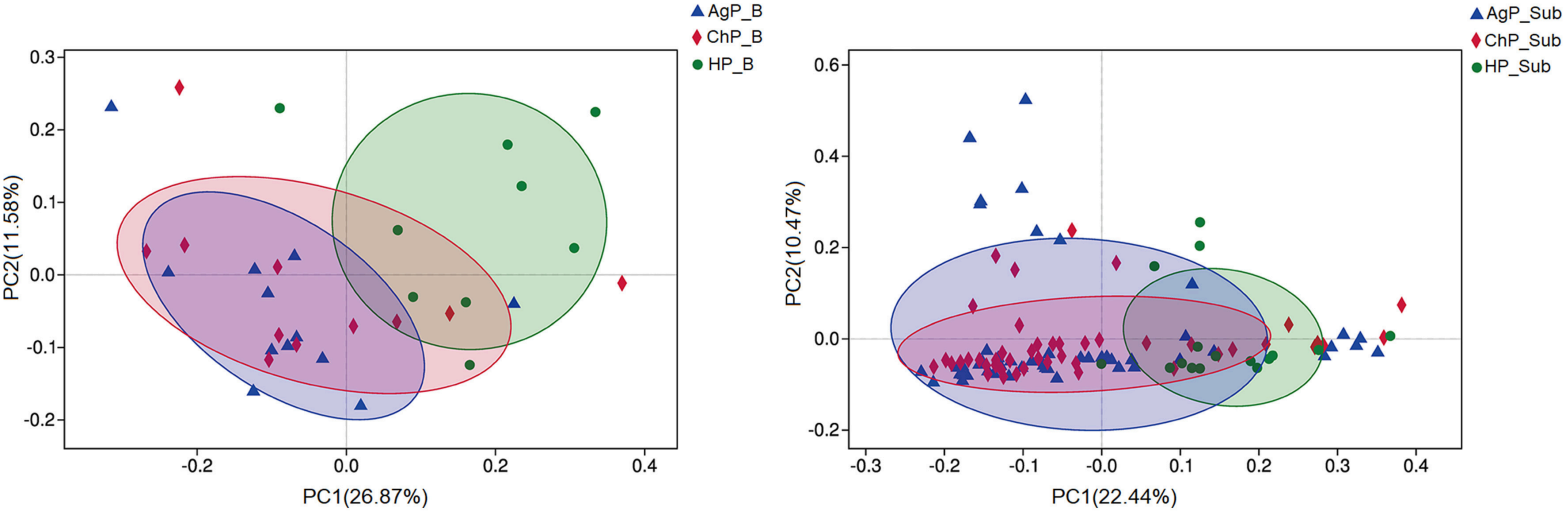
β-diversity of buccal **(Left)** and subgingival plaque **(Right)** samples in three groups. Principal coordinates analysis (PCoA) of unweighted UniFrac distance were performed based on the OTU abundances.

To analyze the distribution of microbiota among various groups, principal coordinates analysis (PCoA) were conducted based on the OTU abundances. The ChP_B and AgP_B samples clustered together and could not be distinguished from each other, while the HP_B samples appeared more dispersive (Figure 5). As for subgingival plaque samples, the trend was in agreement with buccal samples, however all subgingival plaque samples clustered tighter when compared with buccal samples.

**Figure 6:**
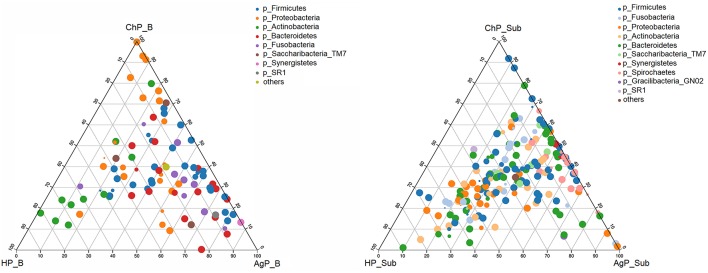
The ternary plots analysis of buccal **(Left)** and subgingival plaque **(Right)** samples on the OTU level which based on three main status: the intraoral environment of periodontal healthy status (HP), and the intraoral environment of periodontitis (ChP/AgP). The dots represented as OTU, the size of the dots represented the OTU abundances and the color represented as different phylum. The OTUs whose abundance <0.01 in all samples were classified into others.

We analyzed our data in phyla level using ternary plots based on three main status: the intraoral environment of periodontal healthy status (HP), and the intraoral environment of periodontitis (ChP and AgP). According to the most abundant OTUs (Figure 6), HP_B harbored a high abundance of Actinobacteria, whereas ChP_B had a high abundance of Proteobacteria and AgP_B had a relatively high abundance of Firmicutes, Bacteroidetes and Synergistetes. Meanwhile, Fusobacteria and Saccharibacteria_TM7 were associated with both ChP_B and AgP_B bacterial communities. As for the subgingival plaque from periodontal pockets, the situation became different and complicated. The diversities increased and OTUs clustered more together between all groups than that in buccal samples, which was consistent with previous PCoA results.

**Figure 7:**
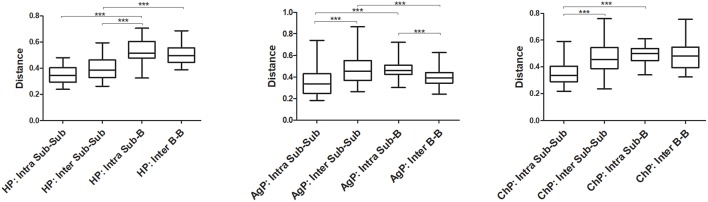
The Unweighted UniFrac distances were calculated and compared between groups to evaluate the similarity of all samples. The distances between subgingival plaque from the same (Intra Sub-Sub) or different individuals (Inter Sub-Sub), the distances between buccal bacterial samples and subgingival plaque from the same individuals (Intra Sub-B) and the distances between buccal bacterial samples from different individuals (Inter B-B) were presented in three groups. ^***^p < 0.001. The error bars indicate mean with standard error.

The Unweighted UniFrac distances were calculated and compared between groups to investigate the similarity of all samples (Figure 7). The distances between buccal bacterial samples and subgingival plaque from the same individuals (Intra Sub-B) were maximal in HP, ChP, and AgP groups. As for subgingival plaque samples, the Unweighted UniFrac distances between samples from the same subjects (Intra Sub-Sub) were significantly lower than those between samples from different individuals in the same groups (Inter Sub-Sub).

**Figure 8:**
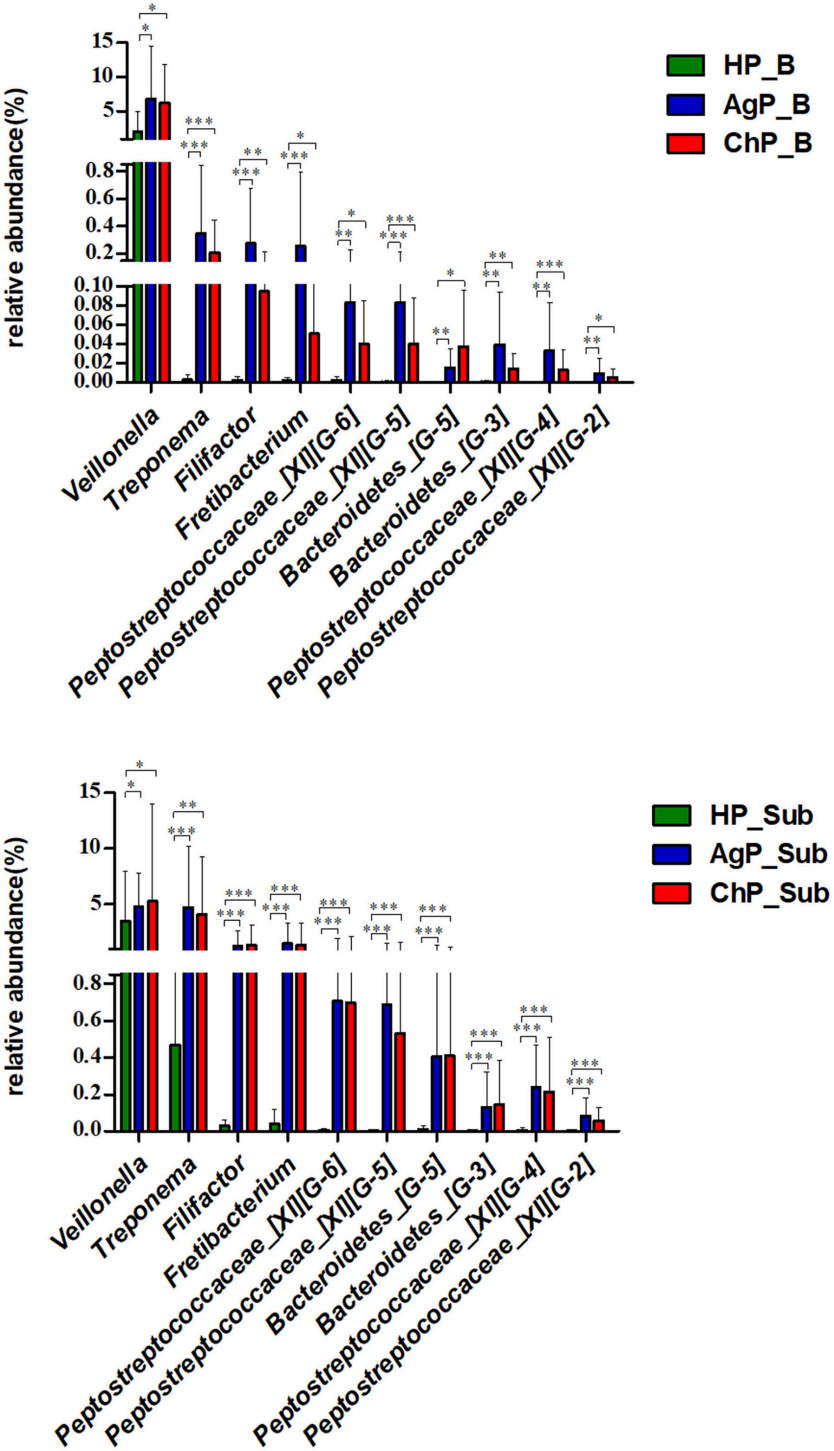
Comparisons of microbiota that presented significantly different contents in both buccal (up) and subgingival plaque (down) samples of periodontitis groups (AgP/ChP) shown the same trend of differences when compared with HP group at genus level. ^*^p < 0.05, ^**^p < 0.01, ^***^p < 0.001.

We compared microbiota between groups at various levels using the Mann-Whitney *U*-test. There were significant differences between all groups at several levels in both buccal and subgingival plaque sample (Supplementary Figures 1–3). The relative abundance of *Veillonella, Treponema, Filifactor, Fretibacterium, Peptostreptococcaceae_[XI][G-6], Peptostreptococcaceae_[XI][G-5], Bacteroidetes_[G-5], Bacteroidetes_[G-3], Peptostreptococcaceae_[XI][G-4]* and *Peptostreptococcaceae_[XI][G-2]* increased significantly in both buccal and subgingival plaque samples of periodontitis groups (ChP and AgP) compared to HP group (Figure 8). However, only *Alloprevotella* had significant difference between ChP and AgP both in two kind of samples.

## Discussion

Periodontitis are polymicrobial infections of the tooth supporting tissues. The primary etiology is plaque biofilm. Periodontitis-associated microorganisms colonize not only subgingival pockets but also other habitats like the buccal mucosa. The current study evaluated the composition of microbiomes on buccal mucosa and subgingival plaques in health and periodontitis using 16S rRNA gene next-generation sequencing analysis. In our current research, we found that higher levels of alpha diversity in buccal and subgingival microbiota of periodontitis patients. While the results also indicated that, along with the changes in periodontal tissue health, the relative abundance of several bacteria would change with them in both two groups of periodontitis and two types of samples. In addition, the results based on the Unweighted UniFrac distance analysis showed that buccal and subgingival plaque samples from the same individuals show higher community divergence than same habitats from different subject samples, which corroborated a notion that individual oral habitats are preferentially populated by different and somewhat unique sets of microbes.

In our research, we used 16S rRNA gene sequencing to evaluate and compare the different characteristics of subgingival plaque and buccal plaque between HP, ChP, and AgP patients. We found that periodontal destruction was associated with higher alpha diversity of microbiota from buccal mucosa samples and subgingival pockets. This may due to the formation and accumulation of dental plaque biofilm, which is the onset of periodontitis; meanwhile, along with the severity and the extent of periodontal tissue destruction, the deepening of the periodontal pockets can provide more spaces and more stable, ideal environment for dental plaque biofilm and bacteria might have the chance to transfer to other oral locations, in our case, buccal mucous.

Previous research reported bacterial community diversity was higher in chronic periodontitis patients than in healthy individuals (Griffen et al., 2012; Abusleme et al., 2013; Camelo-Castillo et al., 2015). It can be considered as the result of a nutritionally richer environment or a reduced immune competence. It is noteworthy that the trend of bacterial shifts accompanying periodontitis is on the opposite situation compared with other polymicrobial infection-related diseases, such as caries (Xiao et al., 2016) or children with black stain (Li et al., 2015) have been associated with a decrease in bacterial diversity. This kind of difference brings up another hint that the progress of periodontitis has relatively special aspects over others. Besides the microbiological reasons, the host defenses and the different environment where the disease occurs may also need to be considered.

Significant differences of oral microbiomes were detected between the health and periodontitis individuals at phyla and genus level in both buccal mucosa and subgingival plaque samples. Segata et al. (2012) reported that over 98% of the buccal mucosa bacteria consisted of 5 main phyla: Firmicutes, Proteobacteria, Bacteroidetes, Actinobacteria, and Fusobacteriaex by exploring the microbiota of 18 body sites (including buccal mucosal surface) in over 200 individuals. In our current study, the bacterial composing of buccal samples from both health and periodontitis corresponded to the results of HMP. While HP_B harbored a high abundance of Actinobacteria, ChP_B had a high abundance of Proteobacteria and AgP_B had a relatively high abundance of Firmicutes, Bacteroidetes, and Synergistetes.

We found 10 genera in both buccal and subgingival plaque samples of periodontitis groups (ChP and AgP) significantly increased when compared with HP group. Among them, *Peptostreptococcus, Treponema*, and *Filifactor* had been reported in previous studies (Kumar et al., 2005; Li et al., 2014; Shi et al., 2015), which were relatively more abundant at the genus level in ChP, and *Treponema* were found at increased abundance in AgP compared with HP (Han et al., 2017). In these genera, some well-known destructive periodontal pathogens were included, such as *Treponema denticola* (*Treponema*) (Socransky et al., 1998). The cluster model of Socransky has been regarded as valid, and the main focus is on the “red” and “orange” complexes that are strongly associated with periodontitis (Socransky et al., 1998). *Micromonas micro*, a member of the genus *Peptostreptococcus*, is a member of the orange complex and is known as a potential pathogen associated with periodontitis. However, the role of some genera in the pathogenesis of periodontitis, such as *Fretibacterium* were not well-studied yet. Based on our current results, when periodontitis occurred in the cavity, the proportion of periodontal pathogens not only increased in subgingival plaques, but also in the samples of buccal plaques. The findings above gave us reason to speculate that some of the periodontal pathogens or opportunistic pathogen could co-aggregate on buccal surfaces or even had the chance to invade into the buccal tissue, as previously shown by other investigators that the *A. actinomycetemcomitans* and *P. gingivalis* both can invade buccal cells (Rudney et al., 2001; Leung et al., 2006).

The PCoA showed all subgingival plaque samples clustered tighter with each other compared with buccal samples. These results agree with those described by Gohler, each oral habitat appears to be preferentially populated by different and somewhat unique sets of microbes (Gohler et al., 2018).

Moreover, the results based on the Unweighted UniFrac distance showed that the distances between buccal swab samples and subgingival plaque from the same individuals (Intra Sub-B) were maximal in each group. The oral microbiota of hard and soft tissues appeared to be characterized by species-specific colonization patterns, which is in accordance with previous study by Mager et al. They reported differences in the bacterial profiles of 40 oral cultivable species on various oral habitats in healthy individuals and found that the profiles of soft tissues were more similar to each other when compared to those from subgingival plaques (Mager et al., 2003). In our study, we presented evidence that fine-scale biogeography variation within the human oral cavity is larger than inter-subject variability in the structure of either subgingival or mucosal communities.

To the best of our knowledge, this is the first study using NGS of the16S RNA gene to characterize the microbiota in two oral habitats (buccal mucosas and subgingival pockets) in patients with ChP or AgP, and to compare them with samples of subjects with periodontal health (HP). We found that buccal and subgingival microbial communities in healthy samples and periodontitis largely differed, with higher abundance in periodontitis. Some genera had significant difference between health and periodontitis both in buccal and subgingival plaque samples. While buccal mucosa and subgingival pockets to be characterized by species-specific colonization patterns.

## Conclusions

In our study, we found that the subgingival and buccal mucosa microbiome in health and periodontitis largely differed, with higher bacterial abundance in periodontitis. In addition, we found 10 genera in both buccal and subgingival plaque samples of periodontitis groups (ChP and AgP) significantly increased when compared with HP group. Moreover, the results based on the Unweighted UniFrac distance showed that buccal and subgingival plaque samples from the same individuals show higher community divergence than same habitats from different subject samples, which corroborated a notion that individual oral habitats are characterized by species-specific colonization patterns. Within the limitation of the relatively small sample size, this pilot study provides a glimpse at the changes in the microbiota of subgingival and buccal mucosa associated with periodontitis from a holistic view.

## Author Contributions

WH and XW: conception and design. YW, MS, MZ, and WH: recruitment of patients and collection of oral samples. MS, YW, CW, and YN: analysis and interpretation of data. YW and MS: manuscript preparation. YW, MS, MZ, WH, and XW: manuscript revisions.

### Conflict of Interest Statement

The authors declare that the research was conducted in the absence of any commercial or financial relationships that could be construed as a potential conflict of interest.
