# Supplementary material for: Comparison of Subgingival and Buccal Mucosa Microbiome in Chronic and Aggressive Periodontitis: A Pilot Study
Source: Front Cell Infect Microbiol. 2019 Mar 11;9:53. doi: 10.3389/fcimb.2019.00053 (PMC6421285; doi:10.3389/fcimb.2019.00053)
Supplement: Supplementary file 1 [file Table_1.DOCX]

**Supplementary Figure 1.** Comparisons of microbiota that presented significantly different contents in buccal (left) or subgingival plaque (right) samples of patients with aggressive periodontitis (AgP) and healthy individuals (HP) at genus level. **p* < 0.05, ***p* < 0.01, ****p* < 0.001.

**Supplementary Figure 2.** Comparisons of microbiota that presented significantly different contents in buccal (left) or subgingival plaque (right) samples of patients with chronic periodontitis (ChP) and healthy individuals (HP) at genus level. **p* < 0.05, ***p* < 0.01, ****p* < 0.001.

**Supplementary Figure 3.** Comparisons of microbiota that presented significantly different contents in buccal (left) or subgingival plaque (right) samples of patients with aggressive periodontitis (AgP) and chronic periodontitis (ChP) at genus level. **p* < 0.05, ***p* < 0.01, ****p* < 0.001.
