# Supplementary figures and images for: Comparison of Subgingival and Buccal Mucosa Microbiome in Chronic and Aggressive Periodontitis: A Pilot Study
Source: Front Cell Infect Microbiol. 2019 Mar 11;9:53. doi: 10.3389/fcimb.2019.00053 (PMC6421285; doi:10.3389/fcimb.2019.00053)

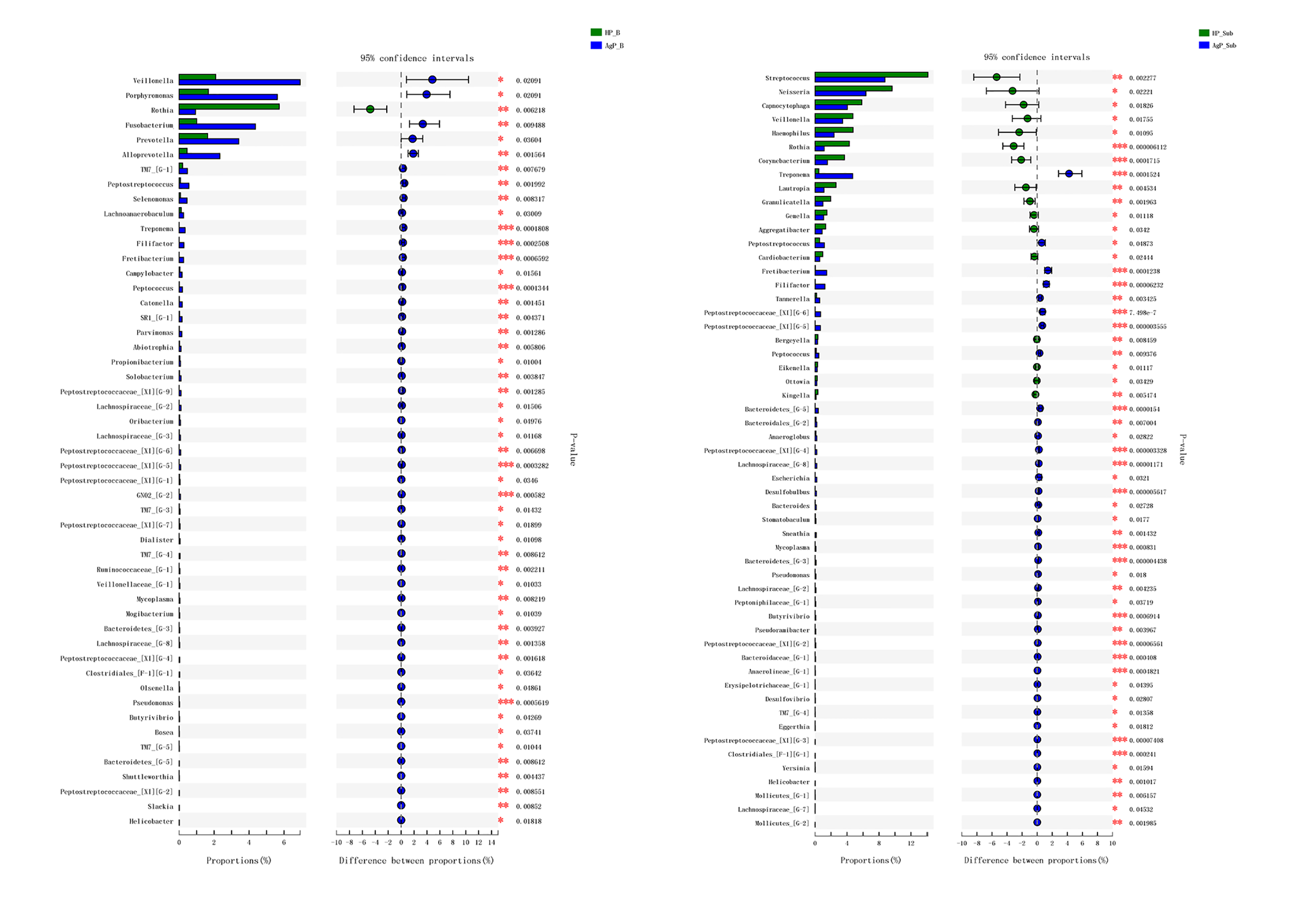

Supplement: Supplementary file 2 [file Image_1.TIF]

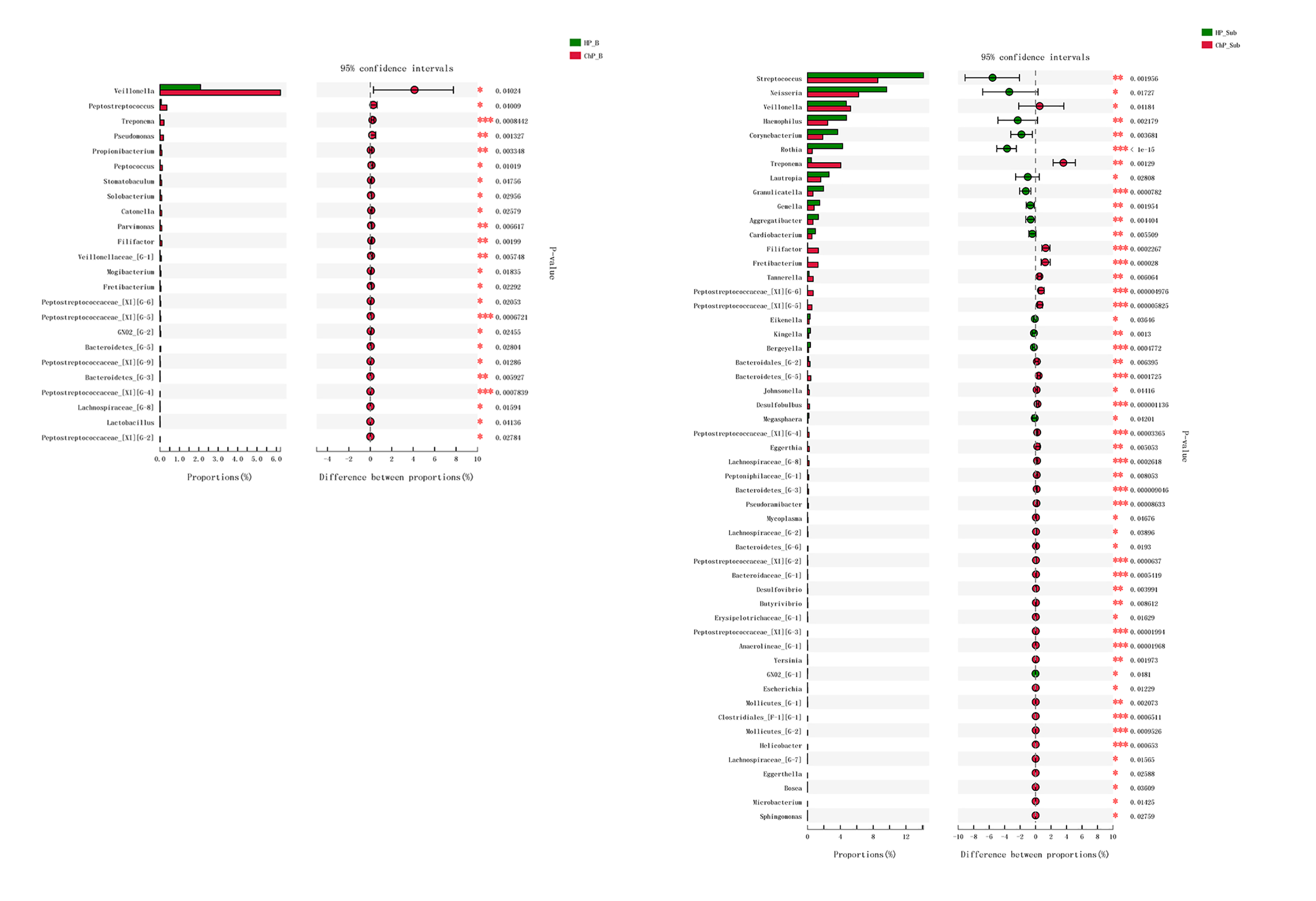

Supplement: Supplementary file 3 [file Image_2.TIF]

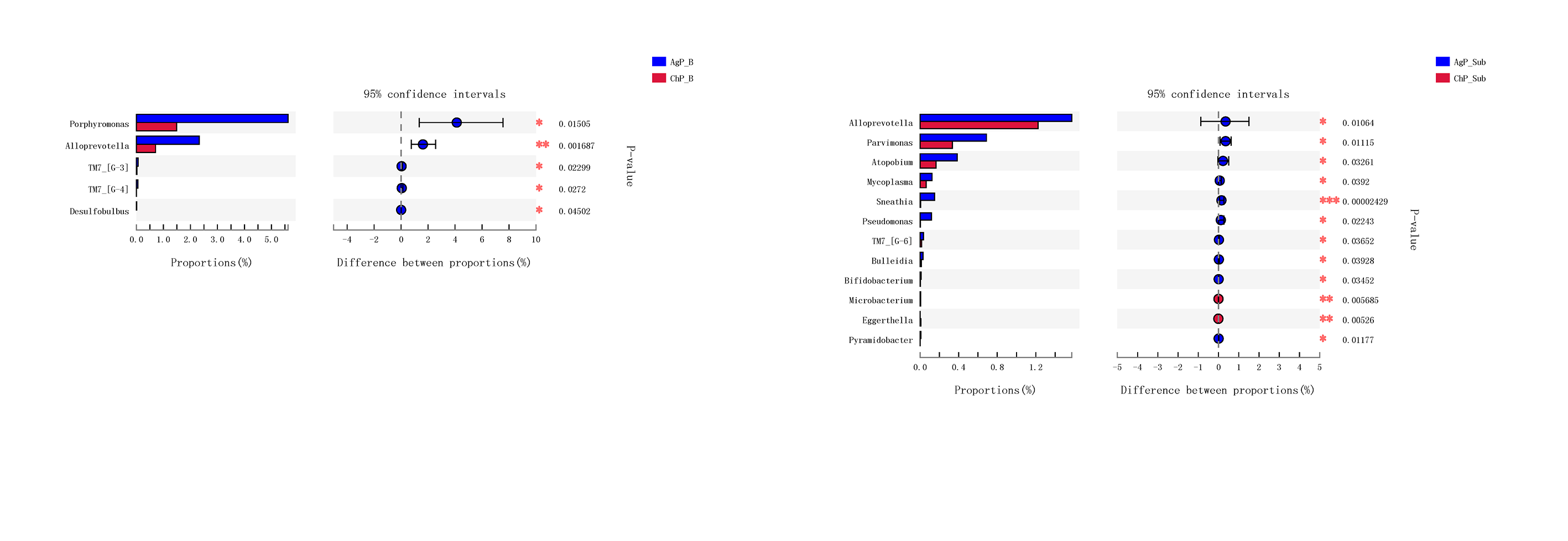

Supplement: Supplementary file 4 [file Image_3.TIF]
